# Effects of a multi-strain probiotic on growth, health, and fecal bacterial flora of neonatal dairy calves

**DOI:** 10.5713/ab.21.0084

**Published:** 2021-08-21

**Authors:** Yongqing Guo, Zheng Li, Ming Deng, Yaokun Li, Guangbin Liu, Dewu Liu, Qihong Liu, Qingshen Liu, Baoli Sun

**Affiliations:** 1 Guangdong Laboratory of Modern Agricultural Science and Technology in Lingnan, South China Agricultural University, Guangzhou 510642, China; 2 College of Animal Science, South China Agricultural University, Guangzhou 510642, China; 3 Jiangsu Hengfengqiang Biotechnology Co., Ltd, Nantong 226121, China

**Keywords:** Calf, Diarrhea Rate, Fecal Bacterial Flora, Performance, Probiotic

## Abstract

**Objective:**

The aim of this study was to investigate the effects of dietary supplementation with a multi-strain probiotic (MSP) product containing of *Bifidobacterium animalis*, *Lactobacillus casei*, *Streptococcus faecalis*, and *Bacillus cerevisiae* on growth, health, and fecal bacterial composition of dairy calves during the first month of life.

**Methods:**

Forty Holstein calves (24 female and 16 male) at 2 d of age were grouped by sex and date of birth then randomly assigned to 1 of 4 treatments: milk replacer supplementation with 0 g (0MSP), 2 g (2MSP), 4 g (4MSP), and 6 g (6MSP) MSP per calf per day.

**Results:**

Supplementation of MSP did not result in any significant differences in parameters of body measurements of calves during the 30 d period. As the dosage of MSP increased, the average daily gain (p = 0.025) and total dry matter intake (p = 0.020) of calves showed a linear increase. The fecal consistency index of the 2MSP, 4MSP, and 6MSP group calves were lower than that of the 0MSP group calves (p = 0.003). As the dosage of MSP increased, the concentrations of lactate dehydrogenase (p = 0.068) and aspartate aminotransferase (p = 0.081) in serum tended to decrease, whereas the concentration of total cholesterol increased quadratically (p = 0.021). The relative abundance of *Dorea* in feces was lower (p = 0.011) in the 2MSP, 4MSP, and 6MSP group calves than that in the 0MSP group calves. The relative abundance of *Dorea* (p = 0.001), *Faecalibacterium* (p = 0.050), and *Mitsuokella* (p = 0.030) decreased linearly, whereas the relative abundance of *Prevotella* tended to increase linearly as the dosage of MSP increased (p = 0.058).

**Conclusion:**

The MSP product can be used to reduce the diarrhea, improve the performance, and alter the composition of the fecal bacteria in neonatal dairy calves under the commercial conditions.

## INTRODUCTION

Calf management is one of the most important tasks in dairy production, which requires a great deal of technology, equipment, and experience. In intensive dairy farm, calves are extremely susceptible to suffering enteric bacteria imbalance or gastrointestinal infection, which can cause lower digestion and absorption of nutrients, delayed growth, even diarrhea and dehydration [1]. The traditional practices employed by dairy farmers to treat diarrhea include using large quantities of antibiotics to combat invading pathogens or using electrolyte therapy to replenish lost fluids [2]. However, there has been great concern about the use of antibiotics which may result in antibiotic resistant pathogens, posing a potential risk of exposure for humans and calves [3]. The use of antibiotic in the feed of livestock was banned in European Union from 2006 and in China from July 1, 2020. Therefore, it is particularly important to find good alternatives to antibiotics, especially in preventing and treating diarrhea in calves and other young animals.

The number of bacteria in the gastrointestinal tract of neonatal calves is relatively small, and the balance of the bacteria can be easily damaged, especially when raising early-weaned calves [4]. Under the guidance of the microecology theory, probiotics represent a kind of living bacterial preparation that produces growth-promoting substances, which is beneficial and harmless to the host [5]. Not only can probiotics reduce the occurrence of diseases, but also are able to improve the feed conversion rate and productive performance of livestock [6,7]. Various bacteria have been used as probiotics in ruminants, mainly including yeast *S. cerevisiae*, *Lactobacillus* spp., *Bifidobacterium* spp., *Propionibacterium* spp. [8]. The combination of *Lactobacillus acidophilus* and *Bifidobacterium* has been found to have positive effects on the immune function of lambs [9]. Probiotics can stimulate the production of mucus by intestinal goblet cells and are essential for protecting the host from colonisation and invasion by pathogens, as well as lubricating the intestinal epithelium [10]. Frizzo et al [11] reported that supplementing with lactic acid bacteria as a feed additive during the preweaning period can reduce the occurrence of diarrhea, improve average daily gain (ADG) and feed efficiency of dairy calves.

Supplementing beneficial bacteria to the feed of pig or poultry is not a new concept [12]. However, how to use live probiotics in the feed of young ruminants is rarely published and the results are inconsistent [13,14]. Therefore, the purpose of the current study was to determine whether supplementing a MSP product with *Bifidobacterium animalis*, *Lactobacillus casei*, *Streptococcus faecalis*, and *Saccharomyces cerevisiae* as a feed additive in milk replacers, can improve the growth, health, antioxidant capacity, and positively influence the microbiota of dairy calves during the first month of life.

## MATERIALS AND METHODS

### Animal care

The experimental design and procedure presented in this study were reviewed and approved by the Animal Care and Use Committee of the South China Agricultural University, Guangzhou, China (approval number SCAU#2013-10).

### Feed additives

An MSP product (Fengqiang Shengtai, Jiangsu Heng-feng-qiang Biotechnology Co., Ltd, Nantong, China) was used in this study. It was composed of equal proportions of four live bacterial strains, namely *Bifidobacterium animalis* (1.0×10^7^ colony-forming unit [CFU]/g), *Lactobacillus casei* (1.0×10^7^ CFU/g), *Streptococcus faecalis* (1.0×10^6^ CFU/g), and *Bacillus cerevisiae* (1.0×10^6^ CFU/g). All the four stains are permitted as probiotics in the registration of ministry of agriculture of China (2014-101357037). The product was stored at 4°C and protected from light.

### Treatments

Forty Holstein calves were divided into four homogenous groups according to sex (24 female and 16 male) and by date of birth. The four treatments differed depending on the feed additives, the four groups were as follows: control (0MSP, without feed additive), and the calves fed with 2 g (2MSP), 4 g (4MSP), and 6 g (6MSP) per calf per day. All experimental feed additives were mixed into the reconstituted milk replacer immediately before morning feeding throughout the study.

### Animals and management

The experiment was conducted at the Zhaoqing Wens dairy farm (Zhaoqing, Guangdong, China). This facility houses approximately 5,300 dairy cattle, 2,800 of which are milking cows. A total of 40 healthy Holstein calves (at 2 d of birth) were all obtained from the same farm. The calves separated from their dams within 2 h of birth and moved to a naturally ventilated barn with individual pens (1.55 m×0.81 m×1.33 m; length×width×height), with leak board and bedded with dry straw for the duration of the trial. Calves were enrolled in the experiment over a period of 1 month from October to November 2019, and which were grouped by date of birth in separate rows for male and female. All calves were born within 24 h on the enrollment date (referred to as age 0 d). The pens were refreshed every 3 days, and manure was removed daily to keep the bottom of pens dry and clean. The temperature of the calf barn was maintained at 21.5°C±3.5°C.

In the first 24 h, calves received 6 L colostrum with a high concentration of immunoglobulins (>50 mg/mL of immunoglobulin G [IgG]). On day 2, calves were fed transition milk (4 L/d divided into 2 equal feedings) at 7:00 AM and 6:00 PM. Calves were fed 4 L from 3 d to 10 d, 6 L from 11 d to 23 d, 8 L from 24 to 30 d of reconstituted milk replacer (220 g/kg crude protein [CP], 150 g/kg ether extract, dry matter [DM] basic, 125 g powder as feed/L, Eurolac Blue, Netherlands) in 2 feedings at 07:00 and 18:00 until they were 30 d of age. The reconstituted milk replacer was heated between 39°C to 42°C before feeding. All calves consumed the same amount of milk replacer and had drinking water freely available. During the experiment, from d 3 to 30, calves were fed *ad libitum* with pelleted starter feed (265.9 g/kg CP, DM basic, Guangdong Wens Dairy Co. Ltd, Guangdong, China). The starter feed was delivered at 9:00 AM to allow at least 10% feed refusals, which were collected and weighed on an individual basis. The nutritional components of milk replacer are presented in Table 1, and the nutrients of starter feed were analyzed according to the Association of Official Analytical Chemists [15].

### Feed intake and growth performance

The amounts of starter feed offered and refused were recorded every day to calculate the individual total dry matter intake (TDMI; milk replacer and starter feed), ADG, and feed efficiency. The skeletal growth (heart girth, withers height, hip height, and hip width) were measured on d 3 and d 30 (before morning feeding) according to the method described by Khan et al [16]. Calves were scored for manure before morning feeding every day. The feces score was determined following Marcondes et al [17], according to a standard scoring procedure (0 = normal feces; 1 = semi-formed feces; 2 = loose feces; and 3 = watery feces) by two students who were blinded to the experimental groups and trained by a veterinary practitioner. Diarrhea was considered when the feces score was ≥2. The onset and duration of diarrhea in all calves were recorded. The diarrhea rate was calculated according to the procedure described by Sun et al [18], and formula is given as: Diarrhea rate = (number of calves with diarrhea × days of diarrhea)/(total number of calves × examined days) × 100. The fecal consistency index (FCI) proposed by Marcondes et al [17] was employed. The FCI was calculated at different stages of the experiment to judge the softness versus hardness of feces, as follows:


$$\text{FCI}=\frac{\left(\text{dE}1\times 1)+(\text{dE}2\times 2)+(\text{dE}3\times 3)+(\text{dE}4\times 4\right)}{\text{Td}\times 4}$$

In the formula, dE1, dE2, dE3, and dE4 represent the days when the feces score was 1, 2, 3, and 4, respectively. Td represents the test evaluation days.

Calf health was monitored daily by a veterinarian unaware of the treatments during the experimental period. Calves of 0MSP with diarrhea were treated with the standard procedure prescribed by the veterinarian, 3 mL 10% oxytetracycline (Eastern Along Pharmaceutical Co., Ltd., Foshan, China) injected intramuscularly before morning feeding. Calves in the 2MSP, 4MSP and 6MSP groups were only fed with MSP, and no drug treatments were administered. No calves had pneumonia or died during the experimental period.

### Blood sampling and analysis

Before morning feeding at 30 days of age, 10 mL of blood was collected by jugular puncture of each calf using a vacuum coagulation-promoting vessel and the blood samples were centrifuged at 3,000 r/min for 15 min. Serum was separated and partitioned into 1.5 mL aliquots and stored at −80°C for further analyses. Test kits developed by Zhongsheng North Holding Biotechnology Co. Ltd (Beijing, China) were used to determine the content of glucose (GLU), total cholesterol (TC), uric acid, urea, creatinine, total protein, globulin, albumin, and the activity of lactate dehydrogenase (LDH), alanine aminotransferase, aspartate aminotransferase (AST), and alkaline phosphatase. The levels of total antioxidant capacity, malonaldehyde (MDA), superoxide dehydrogenase, and glutathione peroxidase in serum were tested with colorimetric assay kits (Nanjing Jiancheng Bioengineering Institute, Jiangsu, China) following the manufacturer’s instructions, using visible recording spectrophotometer (V-T3, Yipu Corp, China). The levels of IgA, IgM, and IgG were tested with enzyme-linked assay kits (Shanghai Ketao Biotechnology Centre, Shanghai, China) following the manufacturer’s instructions, using an enzymatic analyzer (Rayto RT-6100, Rayto Corporation, Shenzhen, China).

### Feces sampling and analysis

The fresh feces of each calf were collected with disposable sterile gloves by stimulating the rectum before morning feeding at 30 days of age. The samples were divided into 2 mL cryopreserved tubes and placed in liquid nitrogen. The samples were taken back to the laboratory and stored at −80°C for analysis of fecal microflora.

Thirty-nine fecal samples (10 samples in each group, except that 9 samples in 2MSP group) were taken out of the refrigerator at −80°C and thawed in ice water, and the whole process of aseptic operation was performed. Total genomic DNA was extracted from fecal samples using an EZNA DNA kit (Omega M5635-02, Norcross, GA, USA) following the manufacturer’s instructions. A NanoDrop ND-1000 System (Thermo Fisher, Wilmington, DE, USA) was used to measure the concentration of DNA. Total DNA was then diluted to a final concentration of 20 ng/μL and stored at −20°C until analysis. The V3–V4 region of the 16S rRNA gene was amplified using the following primers: F (5′-ACTCCTACG GGAGGCAGCA-3′) and R (5′-GGACTACHVG GGTW TCTAAT-3′). The total reaction volume of 25 μL comprised 2 μL of 2.5 mM dNTPs, 5 μL of 5×reaction buffer, 0.25 μL of Q5 DNA polymerase, 1 μL of each primer, 2 μL of DNA template, and 2 μL of ddH_2_O. The real-time polymerase chain reaction (PCR) conditions were 98°C for 2 min, followed by 20 to 35 cycles of denaturation at 98°C for 15 s, and annealing and extension at 55°C for 30 s and at 72°C for 5 min. All samples examined in this study provided complete DNA samples, as agarose gels clearly showed amplified products. After PCR amplification, amplicons were extracted from 2% agarose gels and purified using a Quant-iT PicoGreen dsDNA Assay Kit. Purified amplicons were operated using paired-end sequencing by Illumina MiSeq. The instructions of the platform and the manufacturer were from a commercial service provider (Personalbio, Shanghai, China). To ensure the sequencing quality, the optimal sequencing length of the target fragment was recommended to be 500 bp. All processes were carried out by the commercial service provider.

### Bioinformatics and statistical analysis

The PANDAseq assembler was used to merge and repair overlapping double-ended Illumina fastq files. All sequences with low quality base call scores and sequences with unmodulated bases (N) in the overlapped region were discarded. The fast file was then analyzed by the downstream computing pipeline of QIIME (2.0 version), an open-source software package, using the default minimum quality threshold of 25. The chimerism sequences were detected using UCHIME algorithm (USEARCH 6.1) for ab initio and reference-based chimerism detection. Using the Greengenes database (version 13.5), sequences were clustered at the 97% sequence similarity level using operational taxonomic units (OTUs) collection method based on open reference, the QIIME algorithm and the USEARCH 6.1 method using the default parameters. Sequence quilt samples that failed to cluster were used for ab initio OTU removal. All selected OTUs were then aligned by PyNAST, and the FastTree method was used to construct the phylogenetic tree to calculate the UniFrac distance in QIIME. An RDP classifier was used to assign representative OTU to bacterial classification through QIIME, with a confidence threshold of 0.8.

The data measured in the experiment were preliminarily sorted using Excel 2016 software, and analyzed as a completely randomized design using the generalized linear mixed models procedure of the SAS (version 9.4, SAS Institute Inc., Cary, NC, USA) via the following model: Y_ijk_ = μ+T_i_+S_k_+ɛ_ij_, where Y_ijk_ = the dependent variable value of the test cattle in different treatment groups; μ = population mean; T_i_ = dietary treatment effect (i = 1, 2, 3, 4); S_k_ = sex effect (k = 1,2); and ɛ_ij_ = random error. All the above data were compared with Tukey’s range test, and an orthogonal polynomial model was used to analyse the relationship between the linear increased of MSP and each corresponding index (overall, linear, and quadratic curve). Treatment effects were declared significant at p<0.05, and the tendencies were from p>0.05 to <0.10.

## RESULTS

### Intake and growth performance

There was no significant difference (p>0.05) in the body measurements parameters (ADG of body height, body length, chest girth, and cannon girth) among four treatments (Table 2). The body weight on 30 d (p = 0.071), ADG (p = 0.077), starter feed intake (p = 0.096) and TDMI (p = 0.096) of calves showed an increasing trend, with increasing the dosage of MSP. Meanwhile, the body weight gain (p = 0.085), ADG (p = 0.025), starter feed intake (p = 0.020), and TDMI (p = 0.020) of calves showed linear increase as the dosage of MSP increased.

### Diarrhea incidence

As shown in Table 3, compared with the 0MSP calves, the diarrhea rates of the 2MSP, 4MSP, and 6MSP calves were all reduced, from 70% for 0MSP to 10% 0MSP for 6MSP. The FCI of the 2MSP, 4MSP, and 6MSP calves were lower (p = 0.003) than the control group calves. However, there were no significant difference of FCI among the 2MSP, 4MSP, and 6MSP calves.

### Serum metabolites

As the dosage of MSP increased, the concentrations of LDH (p = 0.068) and AST (p = 0.081) in serum tended to decrease, the concentration of LDH decreased linearly (p = 0.025), and the concentration of AST decreased quadratically (p = 0.015), whereas the concentration of TC increased quadratically (p = 0.021) (Table 4). Moreover, the concentration of TC in serum increased (p = 0.043) significantly in 4MSP calves than the 6MSP calves. The other parameters of serum were not affected by dietary treatment.

### Serum antioxidant capacity and immunological parameters

The treatments did not affect (p>0.05) any antioxidant or immunological parameters in the serum of four groups (Table 5). The contents of MDA were numerically lower (p = 0.130) in the 2MSP, 4MSP, and 6MSP calves than the 0MSP calves.

### Fecal microbiota analysis

**Figure 1:**
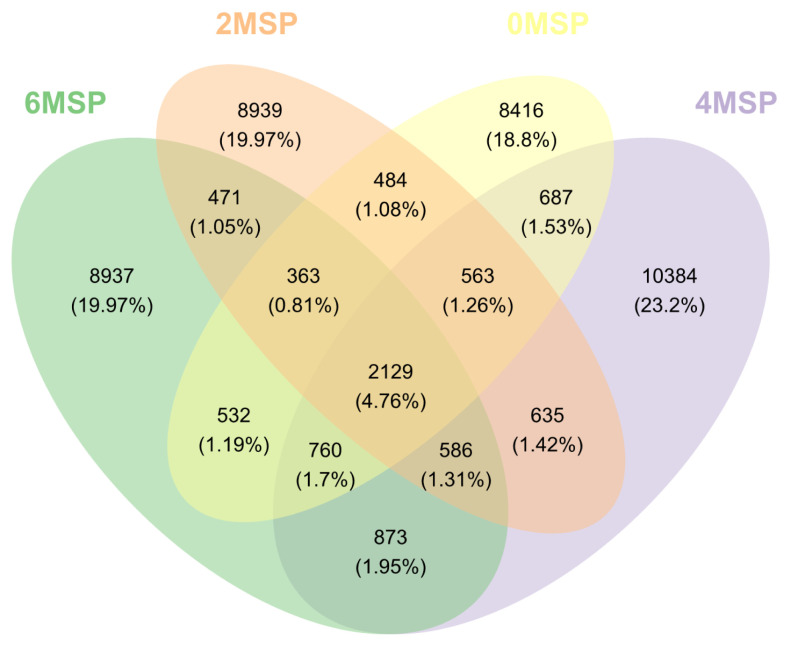
A Venn diagram analysis. A Venn diagram showing shared and unique OTUs at 97% identity among the four groups^1)^. OUT, operational taxonomic units; MSP, multi-strain probiotic. ^1)^ 0MSP, 2MSP, 4MSP, 6MSP: a basal diet with 0, 2, 4, and 6 g MSP per calf per day, respectively.

Diversity of fecal bacterial communities: A total of 5,205,474 sequences were obtained from 39 fecal samples sequenced in the V3–V4 region of the 16S rRNA gene on Illumina Miseq high-throughput sequencing platform, with an average of 133,474 sequences per sample. After the comparison, 4,668,610 valid sequences and 2,658,878 high-quality valid sequences were obtained. According to the 97% similarity, effective sequences were clustered into 44,759 OTUs. As shown in Figure 1, there were 2,129 OTUs in the four groups, accounting for 4.76% of the total. There were 563 OTUs in the 2MSP, 4MSP, and 6MSP calves, accounting for 1.26% of the total. The OTU number and proportion of each group were 13,934 and 31.13% in 0MSP, 14,170 for 31.66% in 2MSP, 16,617 for 37.13% in 4MSP, and 14,651 for 32.73% in 6MSP. The unique OTU numbers of the four groups were 8,416, 8,939, 10,384, and 8,937, respectively.

**Figure 2:**
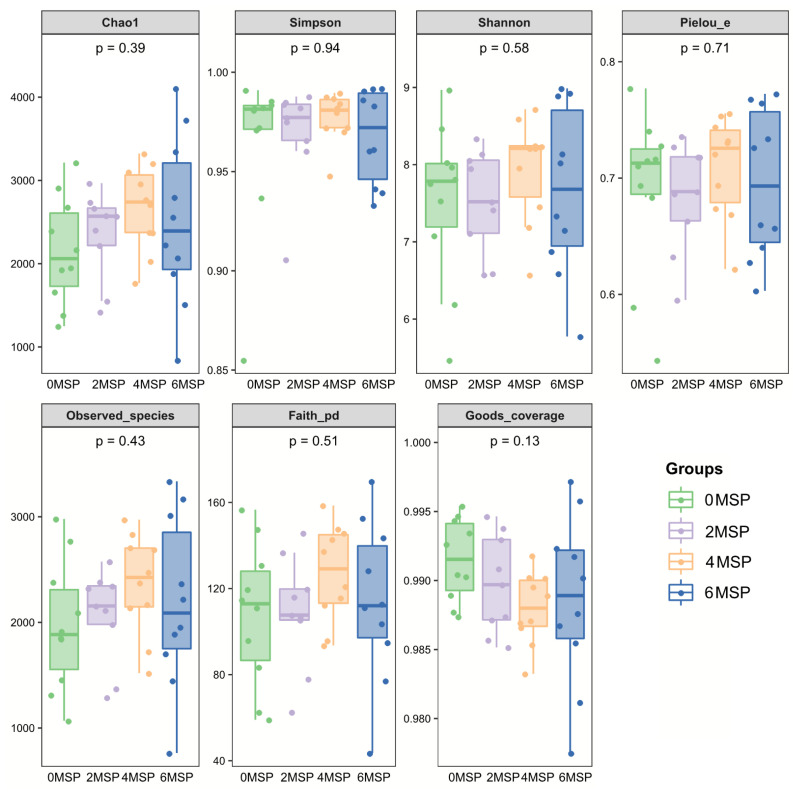
Bentobium plot of the fecal bacterial flora - diversity analysis index^1)^. ^1)^ 0MSP, 2MSP, 4MSP, 6MSP: a basal diet with 0, 2, 4, and 6 g MSP per calf per day, respectively. MSP, multi-strain probiotic.

According to Table 6 and Figure 2, supplementation of MSP had no significant effect on the number of microbial diversity (Shannon, Chaol, Goods coverage, Observed species and Pielou-e). The 4MSP group exhibited the highest value in each index, indicating that the abundance and uniformity of intestinal bacteria in 4MSP were higher than the group 0MSP, 2MSP and 6MSP calves. As the dosage of MSP increased, the Shannon, Chaol and Observed species numerically increased in the fecal microbe.

**Figure 3:**
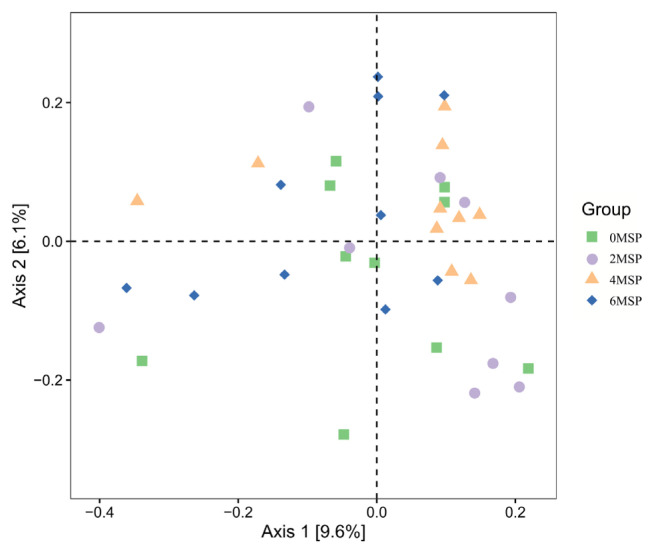
Principal coordinates analysis (PCoA) plots of fecal bacterial flora (Unweighted UniFrac)^1)^. ^1)^ 0MSP, 2MSP, 4MSP, 6MSP: a basal diet with 0, 2, 4, and 6 g MSP per calf per day, respectively. MSP, multi-strain probiotic.

The contributions of the primary coordinates of the first (Axis1) and the second (Axis 2) were 9.6% and 6.1%, respectively (Figure 3). The samples in 6MSP calves were distributed in a wide range, and the degree of polymerisation was relatively low, indicating that the environment of intestinal bacteria was significantly affected by the 6MSP treatment. The samples of 0MSP were mainly distributed in the middle of the coordinate axis. As the dosage of MSP increased, the distribution of 2MSP, 4MSP and 6MSP was shifted, and the distance between 2MSP and 4MSP was relatively close, indicating that the intestinal bacterial flora of these groups was more similar.

#### Fecal bacterial community structure

**Figure 4:**
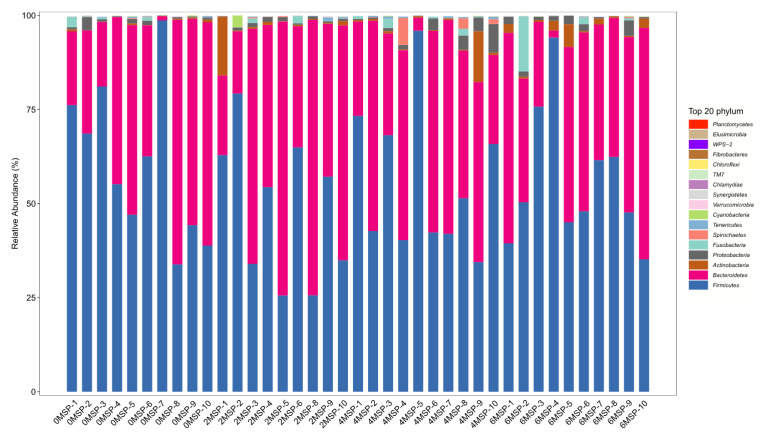
Fecal bacterial flora composition of dairy calves (phylum level, %)^1)^. ^1)^ 0MSP, 2MSP, 4MSP, 6MSP: a basal diet with 0, 2, 4, and 6 g MSP per calf per day, respectively. MSP, multi-strain probiotic.

At the phylum level, a total of 11 phyla had a relative abundance of more than 0.01%, among which four phyla had a relative abundance of more than 1% including *Bacteroidetes*, *Firmicutes*, *Actinobacteria*, and *Proteobacteria*, with a relative abundance of 40%, 55%, 1%, and 1%, respectively (Figure 4). As shown in Table 7, as the dosage of MSP increased, the relative abundance of *Tenericutes* tended to increase (p = 0.092) at the phylum level and increased in a quadratic curve (p = 0.023). However, there were no significant difference regarding the relative abundance of other phyla among the four groups.

**Figure 5:**
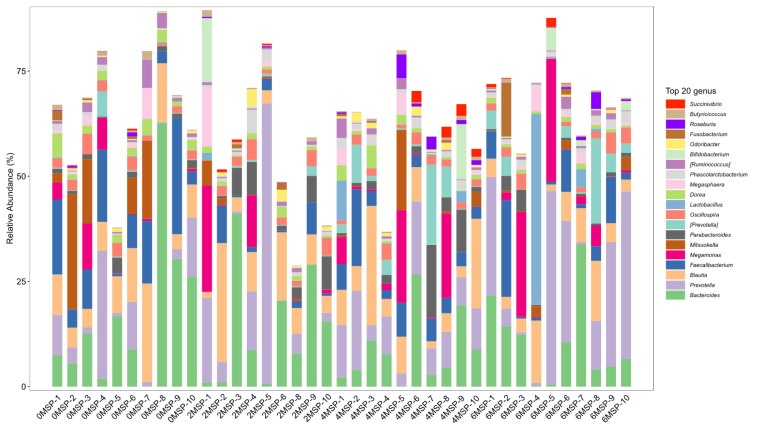
Fecal bacterial flora composition of calves (genus level, %). 0MSP, 2MSP, 4MSP, 6MSP: a basal diet with 0, 2, 4, and 6 g MSP per calf per day, respectively. MSP, multi-strain probiotic.

At the genus level, as shown in Figure 5 and Table 8, the relative abundance of *Dorea* in the 2MSP and 6MSP calves were significantly lower (p = 0.011) than the 0MSP calves, and the relative abundance of *Faecalibacterium* in the 2MSP was significantly lower (p = 0.045) than the 0MSP. As the dosage of MSP increased, the relative abundance of *Dorea* (p = 0.001), *Faecalibacterium* (p = 0.050), and *Mitsuokella* (p = 0.030) decreased linearly, whereas the relative abundance of *Prevotella* tended to increase linearly (p = 0.058).

## DISCUSSION

### Intake and growth performance

The result of the present study indicated that the gut microbiota is closely related to the host health, and the consumption of probiotics has potential beneficial effects on the host [19]. Growth retardation of calves can influence the potential production performance of dairy cows, which is related to feeding management, higher morbidity and mortality, and increased breeding costs [20]. The increased of ADG was due to the higher starter feed and TDMI of MPS calves in our study. Probiotics can produce amylase, cellulase, protease and other substances, which will improve the digestive function of calves and promote their growth [21]. Previous studies have shown that the probiotics can promote the growth of experiencing high incidences of diarrhea calves [22,23], which was similar to our results.

### Diarrhea incidence

The gastrointestinal microflora of newborn calves has not been completely established, which is vulnerable to dietary changes, environmental factors, and other stress, resulting in the destruction of the flora balance, leading to the occurrence of diarrhea [24]. Probiotics entering into the digestive tract of animals can produce various antimicrobial compounds, such as bacteriocins, hydrogen peroxide, volatile fatty acids and nitric oxide, which allows the probiotic bacteria to compete with native or pathogenic species gut bacteria [25]. As a result, the probiotic can enhance the intestinal health by establishing a beneficial gut microbiota and prevent enteric pathogens to infect the intestines of calves [26]. The FCI can reflect the severity of diarrhea in calves. The higher value of FCI means the softer the feces and the more serious of diarrhea. In current study, we found that MSP can effectively prevented diarrhea and reduced the FCI value of calves, indicating that feeding MSP enhanced gut health and inhibited pathogen growth in the gut. The results of our study are consistent with the results of Casper et al [27], who indicated that the fecal scores were improved linearly with increasing *Lactobacillus plantarum* inclusion rate (0, 4, and 8 g/d) in neonatal calves, and probiotics could changes of the intestinal microflora, thereby helping to improve animal intestinal health and growth performance [28].

### Serum metabolites, antioxidant, and immunological parameters

The serum index can be used to evaluate the metabolic capacity of animal nutrients, antioxidant status and the level of immune response [29]. Blood AST level is an important indicator to evaluate liver function, and elevated AST activity indicates liver dysfunction [30]. In the current study, we found that the concentration of AST in serum tended to decrease as the dosage of MPS increased, which means that MSP has the potential to improve the liver function of calves. Under normal physiological conditions, the production and elimination of free radicals in animals are in a dynamic balance, and excessive free radicals will lead to oxidative stress [31]. TC mainly comes from the metabolic process of the liver, and it is one of the important indexes to evaluate liver lipid metabolism. The increased TC concentration of the calves in the current study, which may be related to a better use of the starter feed intake and TDMI by calves fed with high dosage of MSP. Frizzo et al [11] also reported that the young calves fed with lactic acid bacteria diets had a higher serum TC values than fed with control diets. MDA is the product of lipid peroxidation of cell membrane, and its content can indirectly reflect the production of free radicals and the degree of lipid peroxidation of tissues and cells. The content MDA was numerically lower in serum of 2MSP, 4MSP, and 6MSP calves than the 0MSP calves in the current study, which means that the MSP can improve the serum antioxidant capacity, but the effect is limited.

### Microbial composition of feces

The bacterial phyla in the mammalian gastrointestinal tract are mainly composed of *Firmicutes*, *Bacteroidetes*, *Actinomycetes*, and *Proteobacteria*, which are vital to the healthy growth of animals [32]. Our study also found that the dominant phyla in the feces of calves were *Bacteroidetes*, *Firmicutes*, *Actinomycetes*, and *Proteobacteria*, and the relative abundance were 40%, 55%, 1% and 1%, respectively. Diet can affect the presence of *Tenericutes* in the intestinal [33], which tends to be lower in animals feeding with high nutrient diet [34]. With increasing age of animals, the proportion of *Tenericutes* in gut microbiota increased [35]. In the present research, we found that, the relative abundance of *Tenericutes* tended to increase as the dosage of MSP increased, this phenomenon may be explained by the fact that the probiotic could manipulate maturation of intestinal microbial communities and nutrient absorption, which implied that the guts of calves with less abundance of *Tenericutes* was healthier. The dietary MSP did not affect the abundance of others fecal microflora at the phylum level, which in consistent with the results of Ma et al [36], who found that the supplementation of three probiotics, *Saccharomyces cerevisiae*, *Bacillus subtilis*, and *Enterococcus faecalis*, singly or in combination, had no significant effect on the intestinal bacterial abundance or diversity of Sannan dairy goats.

In the current study, we found that the dominant bacteria in the intestinal tract of calves were mainly anaerobic bacteria at the genus level, such as *Bacteroides*, *Prevotella* and *unclassified-clostridiales*, which is consistent with the results of Zhang et al [37]. We also found that dietary MSP stabilized the gut microbiota and reduced the risk of pathogen colonization. *Prevotella* can produce short-chain fatty acids and are able to prevent gastrointestinal tract infection by competing with pathogenic bacteria for binding sites on epithelial cells [21]. *Dorea* and *Mitsuokella* are both rumen digestion bacteria, and *Dorea* is a conditional pathogenic bacteria [38]. The lower *Dorea* and higher *Prevotella* in the feces of calves in our study can explain the reduced of diarrhea rates for calves fed with MSP. Similar to our results, Xu et al [38] also reported that the supplementation of probiotics could decrease the *Dorea* in feces of dairy cows. The relative abundance of *Faecalibacterium* was lower in calves with the addition of MSP in the current study and in healthy calves in the report of Gomez et al [39], mainly because *F. prausnitzii* has been related to anti-inflammatory properties by stimulating the production of anti-inflammatory cytokines, and reducing the secretion of the pro-inflammatory cytokines by the production of butyrate [40].

## CONCLUSION

Overall, this study demonstrated that MSP product can be used in the milk replacer of neonatal dairy calves to reduce diarrhea, improve the ADG and TDMI under the commercial conditions. The relative abundance of beneficial microflora (*Prevotella*) in feces tended to increase, and opportunistic pathogens *Dorea* decreased. The optimal dose was 6 g/d per animal based on the growth performance, diarrhea incidence and microflora in feces under the condition of this experiment.

## Figures and Tables

**Table 1:** Chemical composition of the milk replacer and starter feed

Items	Milk replacer (% as fed)	Starter feed (% DM)
Dry matter	94.80	86.69
Crude protein	22.00	26.59
Ether extract	15.00	ND
Ash	10.00	6.86
Crude fiber	0.20	ND
Calcium	0.90	0.92
Phosphorus	0.60	0.39

DM, dry matter; ND, not determined.

**Table 2:** Effect of MSP on the growth performance of calves

Items	Dietary treatments^1)^				SEM	p-value		
	
	0MSP	2MSP	4MSP	6MSP		All	Linear	Quad
Growth performance								
Initial body weight (kg)	39.18	40.78	37.78	38.48	0.65	0.220	0.291	0.829
Final body weight (kg)	49.80	53.26	49.93	51.34	0.77	0.071	0.847	0.494
Body weight gain (kg)	10.62	12.48	12.15	12.86	0.43	0.254	0.085	0.371
ADG^2)^ (g/d)	382.86	449.29	451.79	462.86	15.53	0.077	0.025	0.239
Feed intake								
Starter feed intake (g/d)	120.53	151.61	161.45	167.41	7.24	0.096	0.020	0.370
Milk replacer intake (g/d)	660.71	660.71	660.71	660.71	0.00			
TDMI^3)^ (g/d)	781.24	812.32	822.16	828.12	7.24	0.096	0.020	0.370
Feed efficiency^4)^	0.48	0.54	0.53	0.54	0.02	0.699	0.315	0.496
Body measurements								
Body height								
Initial (cm)	75.93	79.46	75.13	77.18	0.67	0.109	0.920	0.568
Final (cm)	82.53	84.42	81.57	82.66	0.62	0.431	0.656	0.746
ADG (cm/d)	0.24	0.18	0.23	0.20	0.01	0.420	0.601	0.672
Body length								
Initial (cm)	69.90	69.36	66.42	66.84	0.62	0.109	0.030	0.689
Final (cm)	77.04	77.66	74.05	74.80	0.66	0.154	0.078	0.960
ADG (cm/d)	0.26	0.30	0.27	0.28	0.02	0.836	0.669	0.657
Chest girth								
Initial (cm)	77.47	71.19	77.18	79.98	0.74	0.201	0.936	0.509
Final (cm)	86.38	88.91	87.96	88.17	0.55	0.437	0.377	0.301
ADG (cm/d)	0.32	0.28	0.39	0.33	0.02	0.350	0.466	0.867
Cannon girth								
Initial (cm)	10.52	10.66	10.53	10.53	0.10	0.957	0.914	0.736
Final (cm)	10.86	11.16	11.01	10.91	0.09	0.656	1.000	0.272
ADG (cm/d)	0.01	0.02	0.02	0.01	0.00	0.430	0.780	0.111

MSP, multi-strain probiotic; SEM, standard error of the mean; ADG, average daily gain; TDMI, total dry matter intake.

1) 0MSP, 2MSP, 4MSP, 6MSP: a basal diet with 0, 2, 4, and 6 g MSP per calf per day, respectively.

2) ADG = weight gain/number of days on feed.

3) TDMI, total dry matter intake of milk replacer and starter feed (g/d).

4) Feed efficiency = g ADG/g TDMI.

**Table 3:** Effect of MSP on the diarrhea incidence of calves

Items	Dietary treatments^1)^				SEM	p-value

	0MSP	2MSP	4MSP	6MSP		
Diarrhea rate (%)	70.00	20.00	30.00	10.00	13.15	-
FCI	0.28^a^	0.26^b^	0.26^b^	0.26^b^	0.00	0.003

MSP, multi-strain probiotic; SEM, standard error of the mean; FCI, fecal consistency index.

1) 0MSP, 2MSP, 4MSP, 6MSP: a basal diet with 0, 2, 4, and 6 g MSP per calf per day, respectively.

a,b Mean values within a row with different superscripts differ significantly (Tukey’s test; p<0.05).

**Table 4:** Effect of MSP on the serum biochemical indexes of calves

Items	Dietary treatments				SEM	p-value		
	
	0MSP	2MSP	4MSP	6MSP		All	Linear	Quad
GLU (mmol/L)	4.10	3.79	4.07	3.82	0.10	0.596	0.537	0.881
TC (mmol/L)	2.40^ab^	2.57^ab^	3.03^a^	2.30^b^	0.10	0.043	0.863	0.021
UA (μmol/L)	56.23	53.13	52.83	55.13	1.75	0.895	0.826	0.462
UREA (mmol/L)	3.62	3.64	3.16	3.76	0.12	0.343	0.956	0.247
CR (μmol/L)	73.04	83.34	72.64	70.54	2.28	0.148	0.335	0.146
TP (g/L)	56.99	55.10	56.51	57.94	0.85	0.692	0.577	0.333
ALB (g/L)	26.88	26.49	25.85	26.60	0.37	0.817	0.674	0.470
GLOB (g/L)	30.11	28.61	30.66	31.34	1.05	0.828	0.551	0.613
LDH (U/L)	938.49	802.99	819.09	778.69	24.74	0.068	0.025	0.273
ALT (U/L)	7.62	6.34	7.16	7.31	0.27	0.341	0.962	0.168
AST (U/L)	45.42	36.01	36.72	42.02	1.49	0.081	0.464	0.015
ALP (U/L)	195.38	197.35	267.75	148.46	17.99	0.099	0.634	0.072

MSP, multi-strain probiotic; SEM, standard error of the mean; GLU, glucose; TC, total cholesterol; UA, uric acid; UREA, urea; CR, creatinine; TP, total protein; ALB, albumin; GLOB, globulin; LDH, lactate dehydrogenase; ALT, alanine aminotransferase; AST, aspartate aminotransferase; ALP, alkaline phosphatase.

1) 0MSP, 2MSP, 4MSP, 6MSP: a basal diet with 0, 2, 4, and 6 g MSP per calf per day, respectively.

a,b Mean values within a row with different superscripts differ significantly (Tukey’s test; p<0.05).

**Table 5:** Effect of MSP on the serum antioxidant index of calves

Items	Dietary treatments^1)^				SEM	p-value		
	
	0MSP	2MSP	4MSP	6MSP		All	Linear	Quad
GSH-Px (U/mL)	161.48	159.00	148.87	167.66	5.67	0.504	0.831	0.234
SOD (U/mL)	52.34	56.10	54.99	53.51	2.67	0.949	0.912	0.589
T-AOC (U/mL)	13.50	14.31	12.89	16.20	0.77	0.260	0.227	0.310
MDA (nmol/mL)	5.52	4.49	5.06	5.00	0.10	0.130	0.466	0.110
IgA (μg/mL)	3,395.56	3,472.93	3,523.40	3,331.33	76.74	0.834	0.842	0.399
IgG (mg/mL)	15.75	16.20	16.89	16.67	0.49	0.854	0.444	0.742
IgM (μg/mL)	1,748.88	1,716.88	1,688.83	1,703.82	55.65	0.985	0.754	0.840

MSP, multi-strain probiotic; SEM, standard error of the mean; GSH-Px, glutathione peroxidase; SOD, superoxide dehydrogenase; T-AOC, total antioxidative capacity; MDA, malonaldehyde; Ig, immunoglobulin.

1) 0MSP, 2MSP, 4MSP, 6MSP: a basal diet with 0, 2, 4, and 6 g MSP per calf per day, respectively.

**Table 6:** Effect of MSP on the community diversity (Alpha diversity) of fecal bacterial community of calves

Items	Dietary treatments^1)^				SEM	p-value		
	
	0MSP	2MSP	4MSP	6MSP		All	Linear	Quad
Shannon	7.53	7.44	7.94	7.67	0.14	0.601	0.956	0.736
Chao1	2,154.22	2,313.33	2,661.79	2,506.75	113.46	0.408	0.481	0.491
Pielou-e	0.69	0.68	0.71	0.70	0.01	0.687	0.866	0.916
Observed species	1,967.70	2,025.04	2,360.86	2,186.93	96.68	0.451	0.700	0.543
Goods coverage	0.99	0.99	0.99	0.99	0.00	0.251	0.239	0.391

MSP, multi-strain probiotic; SEM, standard error of the mean.

1) 0MSP, 2MSP, 4MSP, 6MSP: a basal diet with 0, 2, 4, and 6 g MSP per calf per day, respectively.

**Table 7:** Effect of MSP on the relative abundance of fecal bacteria flora of calves (phylum level; %)

Phylum name	Dietary treatments^1)^				SEM	p-value		
	
	0MSP	2MSP	4MSP	6MSP		All	Linear	Quad
*Actinobacteria*	0.27	2.15	1.71	1.72	0.53	0.642	0.330	0.400
*Bacteroidetes*	37.41	48.28	38.31	38.85	3.04	0.655	0.630	0.444
*Cyanobacteria*	0.04	0.38	0.05	0.04	0.08	0.394	0.652	0.282
*Firmicutes*	60.63	47.55	55.64	55.94	3.06	0.596	0.455	0.322
*Fusobacteria*	0.43	0.26	0.55	1.70	0.38	0.567	0.324	0.412
*Proteobacteria*	0.86	0.87	2.09	1.44	0.24	0.263	0.819	0.512
*Spirochaetes*	0.05	0.08	1.13	0.05	0.20	0.134	0.533	0.157
*Tenericutes*	0.13	0.22	0.31	0.07	0.03	0.092	0.389	0.023
*Verrucomicrobia*	0.03	0.03	0.00	0.01	0.01	0.527	0.550	0.743
*Unclassified-bacteria*	0.12	0.14	0.16	0.14	0.01	0.823	0.765	0.580
*Unidentified-bacteria*	0.02	0.03	0.05	0.04	0.01	0.347	0.473	0.228

MSP, multi-strain probiotic; SEM, standard error of the mean.

1) 0MSP, 2MSP, 4MSP, 6MSP: a basal diet with 0, 2, 4, and 6 g MSP per calf per day, respectively.

**Table 8:** Effect of MSP on the relative abundance of fecal bacteria flora of calves (genus level; %)

Genus name	Dietary treatments^1)^				SEM	p-value		
	
	0MSP	2MSP	4MSP	6MSP		All	Linear	Quad
*Bifidobacterium*	0.05	1.85	1.42	0.98	0.52	0.697	0.514	0.319
*Bacteroides*	17.22	14.08	8.69	10.88	2.14	0.515	0.477	0.540
*Prevotella*	7.50	13.59	9.58	19.01	2.36	0.273	0.058	0.708
*unidentified-S24-7*	2.83	6.04	3.90	0.53	1.41	0.639	0.720	0.282
*unidentified-bacteroidales*	6.54	7.27	5.87	1.61	1.23	0.269	0.173	0.260
*Dorea*	2.54^a^	1.07^b^	1.59^ab^	0.82^b^	0.21	0.011	0.001	0.338
*Faecalibacterium*	10.57^a^	2.09^b^	5.62^ab^	5.84^ab^	1.08	0.045	0.050	0.035
*unidentified-ruminococcaceae*	6.09	5.30	3.47	2.81	0.69	0.326	0.209	0.963
*Megamonas*	2.37	3.86	5.19	6.37	1.33	0.727	0.368	0.955
*Mitsuokella*	7.26	0.79	2.36	0.88	1.01	0.106	0.030	0.233
*unidentified-clostridiales*	7.12	11.74	11.44	10.85	1.06	0.376	0.222	0.221

MSP, multi-strain probiotic; SEM, standard error of the mean.

1) 0MSP, 2MSP, 4MSP, 6MSP: a basal diet with 0, 2, 4, and 6 g MSP per calf per day, respectively.

a,b Mean values within a row with different superscripts differ significantly (Tukey’s test; p<0.05).

## References

[1] Lyoo K, Jung M, Yoon S, Kim HK, Jeong DG (2018). Identification of canine norovirus in dogs in South Korea. BMC Vet Res.

[2] Lorenz I, Mee JF, Earley B, More SJ (2011). Calf health from birth to weaning. I. General aspects of disease prevention. Ir Vet J.

[3] Kelsey AJ, Colpoys JD (2018). Effects of dietary probiotics on beef cattle performance and stress. J Vet Behav.

[4] Malmuthuge N, Griebel PJ, Guan LL (2015). The gut microbiome and its potential role in the development and function of newborn calf gastrointestinal tract. Front Vet Sci.

[5] Wang Y, Gong L, Wu YP, Cui ZW, Li WF (2019). Oral administration of Lactobacillus rhamnosus GG to newborn piglets augments gut barrier function in pre-weaning piglets. J Zhejiang Univ Sci B.

[6] Frizzo LS, Zbrun MV, Soto LP, Signorini ML (2011). Effects of probiotics on growth performance in young calves: A meta-analysis of randomized controlled trials. Anim Feed Sci Technol.

[7] Saleem AM, Zanouny AI, Singer AM (2017). Growth performance, nutrients digestibility, and blood metabolites of lambs fed diets supplemented with probiotics during pre- and post-weaning period. Asian-Australas J Anim Sci.

[8] Seo JK, Kim S, Kim MH, Upadhaya SD, Kam DK, Ha JK (2010). Direct-fed microbials for ruminant animals. Asian-Australas J Anim Sci.

[9] Santillo A, Annicchiarico G, Caroprese M, Marino R, Sevi A, Albenzio M (2012). Probiotics in milk replacer influence lamb immune function and meat quality. Animal.

[10] Do Carmo MS, Itapary Dos Santos C, Araújo MC, Girón JA, Fernandes ES, Monteiro-Neto V (2018). Probiotics, mechanisms of action, and clinical perspectives for diarrhea management in children. Food Funct.

[11] Frizzo LS, Soto LP, Zbrun MV (2010). Lactic acid bacteria to improve growth performance in young calves fed milk replacer and spray-dried whey powder. Anim Feed Sci Technol.

[12] Taras D, Vahjen W, Simon O (2007). Probiotics in pigs—modulation of their intestinal distribution and of their impact on health and performance. Livest Sci.

[13] Lutful Kabir SM (2009). The role of probiotics in the poultry industry. Int J Mol Sci.

[14] Alawneh JI, Barreto MO, Moore RJ (2020). Systematic review of an intervention: the use of probiotics to improve health and productivity of calves. Prev Vet Med.

[15] AOAC (1990). Official methods of analysiss.

[16] Khan MA, Lee HJ, Lee WS (2007). Starch source evaluation in calf starter: I. feed consumption, body weight gain, structural growth, and blood metabolites in holstein calves. J Dairy Sci.

[17] Marcondes MI, Pereira TR, Chagas JCC (2016). Performance and health of Holstein calves fed different levels of milk fortified with symbiotic complex containing pre -and probiotics. Trop Anim Health Prod.

[18] Sun YY, Li J, Meng QS, Wu DL, Xu M (2019). Effects of butyric acid supplementation of acidified milk on digestive function and weaning stress of cattle calves. Livest Sci.

[19] Marchesi JR, Adams DH, Fava F (2016). The gut microbiota and host health: a new clinical frontier. Gut.

[20] Sato T, Hidaka Y, Kamimura S (2010). Sugar supplementation stimulates growth performance in calves with growth retardation. J Vet Med Sci.

[21] Cangiano LR, Yohe TT, Steele MA, Renaud DL (2020). Invited Review: Strategic use of microbial-based probiotics and prebiotics in dairy calf rearing. Appl Anim Sci.

[22] Zhang L, Jiang X, Liu X (2019). Growth, health, rumen fermentation, and bacterial community of Holstein calves fed Lactobacillus rhamnosus GG during the preweaning stage. J Anim Sci.

[23] Timmerman HM, Mulder L, Everts H (2005). Health and growth of veal calves fed milk replacers with or without probiotics. J Dairy Sci.

[24] Bendali F, Sanaa M, Bichet H, Schelcher F (1999). Risk factors associated with diarrhoea in newborn calves. Vet Res.

[25] Chenoll E, Casinos B, Bataller E (2011). Novel probiotic Bifidobacterium bifidum CECT 7366 strain active against the pathogenic bacterium Helicobacter pylori. Appl Environ Microbiol.

[26] Uyeno Y, Shigemori S, Shimosato T (2015). Effect of probiotics/prebiotics on cattle health and productivity. Microbes Environ.

[27] Casper DP, Hultquist KM, Acharya IP (2021). Lactobacillus plantarum GB LP-1 as a direct-fed microbial for neonatal calves. J Dairy Sci.

[28] Jiang Z, Wei S, Wang Z (2015). Effects of different forms of yeast Saccharomyces cerevisiae on growth performance, intestinal development, and systemic immunity in early-weaned piglets. J Anim Sci Biotechnol.

[29] Jiao LF, Ke YL, Xiao K, Song ZH, Hu CH, Shi B (2015). Effects of cello-oligosaccharide on intestinal microbiota and epithelial barrier function of weanling pigs. J Anim Sci.

[30] Gu XL, Li H, Song ZH, Ding YN, He X, Fan ZY (2019). Effects of isomaltooligosaccharide and Bacillus supplementation on sow performance, serum metabolites, and serum and placental oxidative status. Anim Reprod Sci.

[31] Lykkesfeldt J, Svendsen O (2007). Oxidants and antioxidants in disease: oxidative stress in farm animals. Vet J.

[32] Shanks OC, Kelty CA, Archibeque S (2011). Community structures of fecal bacteria in cattle from different animal feeding operations. Appl Environ Microb.

[33] Buzoianu SG, Walsh MC, Rea MC (2012). High-throughput sequence-based analysis of the intestinal microbiota of weanling pigs fed genetically modified MON810 maize expressing Bacillus thuringiensis Cry1Ab (Bt maize) for 31 days. Appl Environ Microb.

[34] Ji Y, Kong X, Li H, Zhu Q, Guo Q, Yin Y (2017). Effects of dietary nutrient levels on microbial community composition and diversity in the ileal contents of pregnant Huanjiang mini-pigs. Plos One.

[35] Niu Q, Li P, Hao S (2015). Dynamic distribution of the gut microbiota and the relationship with apparent crude fiber digestibility and growth stages in pigs. Sci Rep-Uk.

[36] Ma ZZ, Cheng YY, Wang SQ, Ge JZ, Shi HP, Kou JC (2020). Positive effects of dietary supplementation of three probiotics on milk yield, milk composition and intestinal flora in Sannan dairy goats varied in kind of probiotics. J Anim Physiol Anim Nutr.

[37] Zhang J, Xu C, Huo D, Hu Q, Peng Q (2017). Comparative study of the gut microbiome potentially related to milk protein in Murrah buffaloes (Bubalus bubalis) and Chinese Holstein cattle. Sci Rep-Uk.

[38] Xu H, Huang W, Hou Q (2017). The effects of probiotics administration on the milk production, milk components and fecal bacteria microbiota of dairy cows. Sci Bull (Beijing).

[39] Gomez DE, Arroyo LG, Costa MC, Viel L, Weese JS (2017). Characterization of the fecal bacterial microbiota of healthy and diarrheic dairy calves. J Vet Intern Med.

[40] Foditsch C, Santos TM, Teixeira AG (2014). Isolation and characterization of Faecalibacterium prausnitzii from calves and piglets. Plos One.

